# Supplementary figures and images for: Discrete distributional differential expression (D3E) - a tool for gene expression analysis of single-cell RNA-seq data
Source: BMC Bioinformatics. 2016 Feb 29;17:110. doi: 10.1186/s12859-016-0944-6 (PMC4772470; doi:10.1186/s12859-016-0944-6)

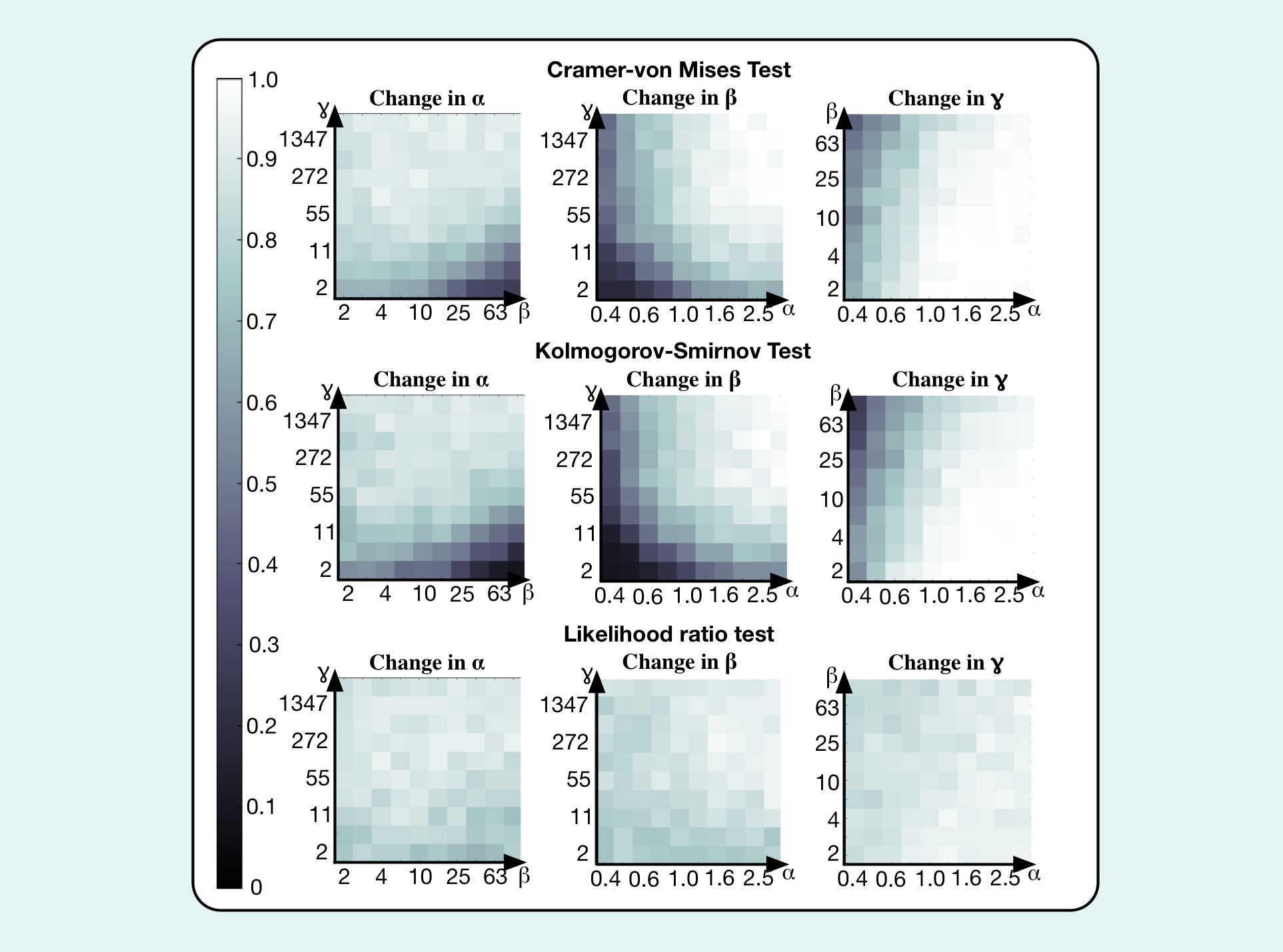

Supplement: Additional file 1 — Figure S1. Sensitivity to changes in parameters of the Poisson-Beta distribution for the Cramér-von Mises, KS and likelihood ratio tests similar to Fig. 4. The range over which the parameters are varied is larger here. The increased presence of lighter colors shows that for the most part it is easier to identify DE genes for this set of parameters. (JPEG 246 kb) [file 12859_2016_944_MOESM1_ESM.jpeg]

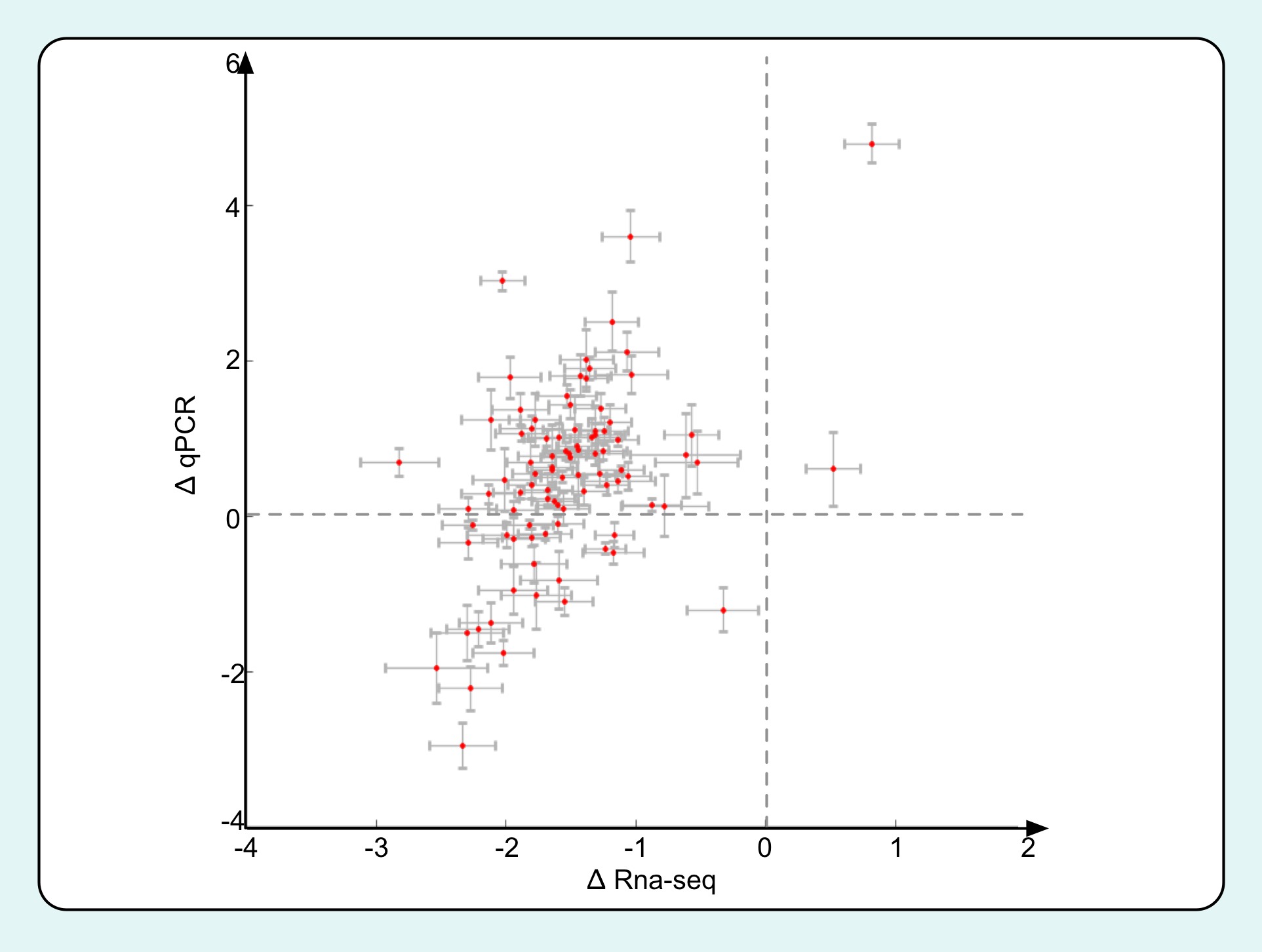

Supplement: Additional file 2 — Figure S2. Change in expression levels for 90 genes from the 2-cell and 4-cell mouse embryos as quantified using either qPCR or RNA-seq [6]. (JPEG 113 kb) [file 12859_2016_944_MOESM2_ESM.jpeg]

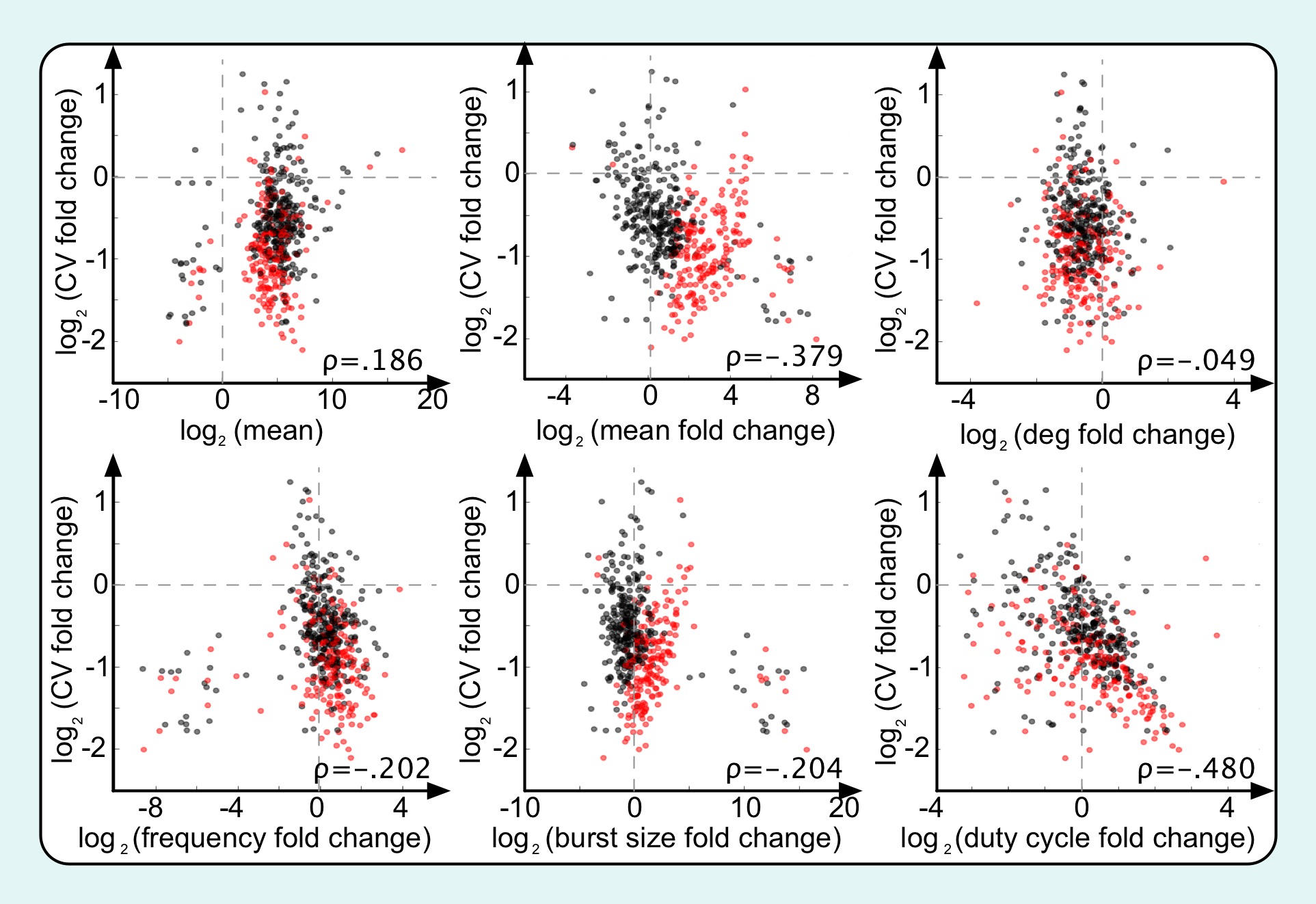

Supplement: Additional file 6 — Figure S3. Scatterplots showing the mean fold-change, as well as the fold-change of the CV compared to the change in degradation rate, burst frequency, duty cycle, burst size. In all panels, black dots represent genes which did not change, red dots represent genes which were deemed significant by D3E. (JPEG 355 kb) [file 12859_2016_944_MOESM6_ESM.jpeg]

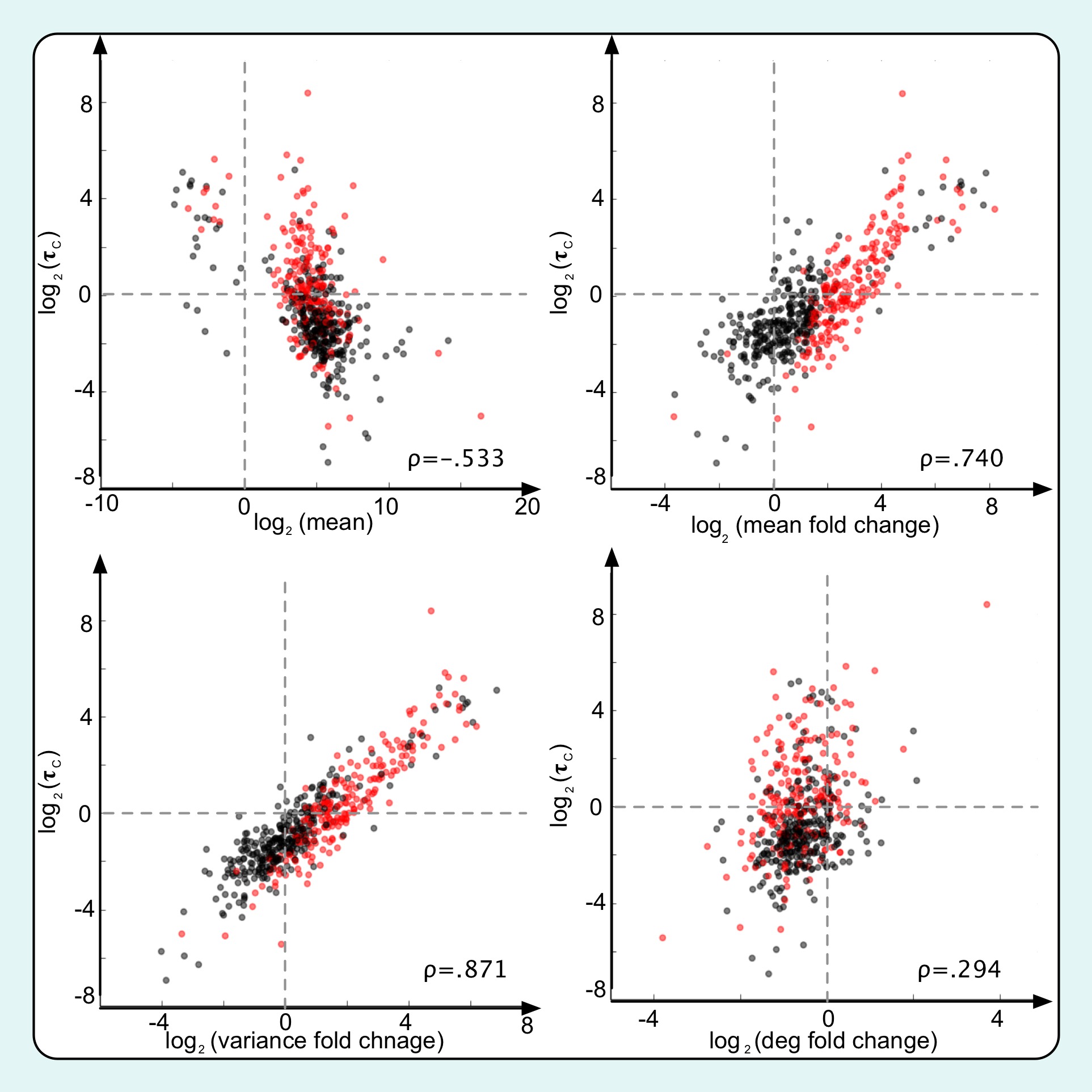

Supplement: Additional file 7 — Figure S4. Scatterplots showing the mean fold-change and the fold-change of the characteristic time, as well as the fold-change of the characteristic time compared to the change in degradation rate, variance and characteristic promoter time. In all panels, black dots represent genes which did not change, red dots represent genes which were deemed significant by D3E. (JPEG 301 kb) [file 12859_2016_944_MOESM7_ESM.jpeg]

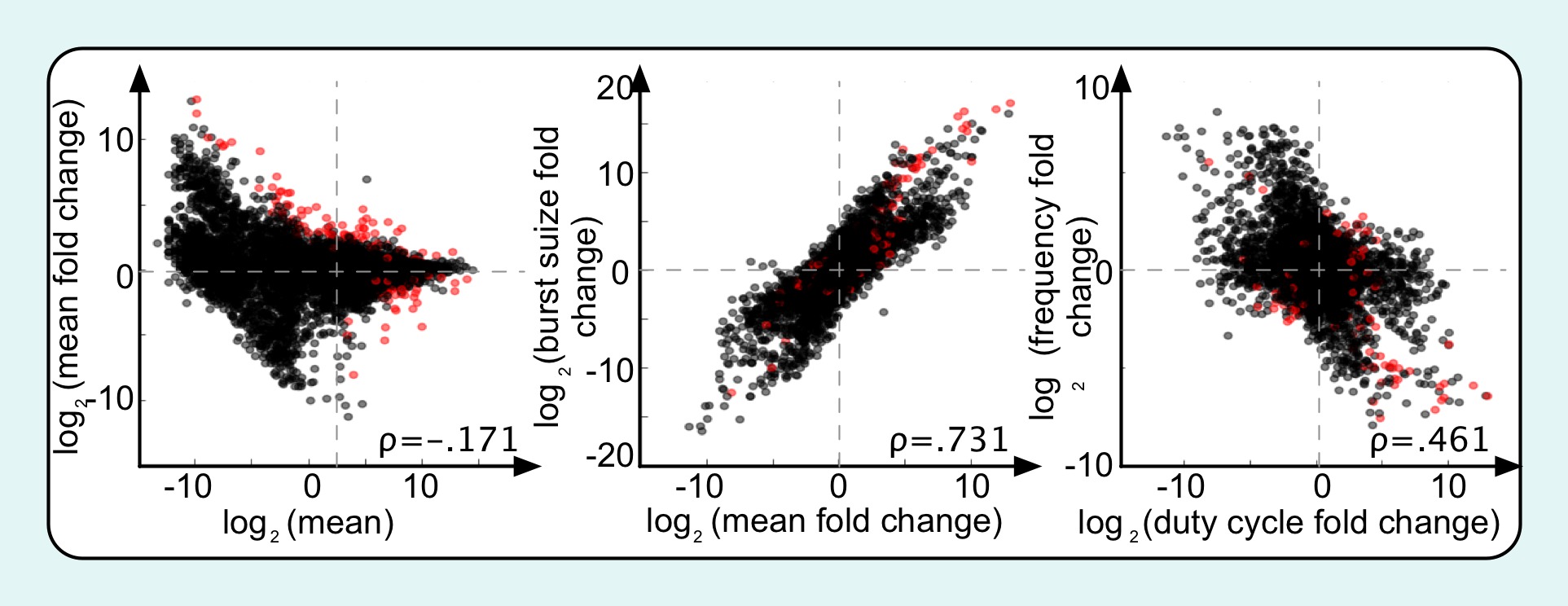

Supplement: Additional file 8 — Figure S5. Scatterplots showing the mean fold-change, as well as the fold-change of the mean compared to the change in burst frequency, duty cycle, burst size for early vs late blastocysts from Deng et al. [10]. In all panels, black dots represent genes which did not change, red dots represent genes which were deemed significant by D3E. (JPEG 189 kb) [file 12859_2016_944_MOESM8_ESM.jpeg]

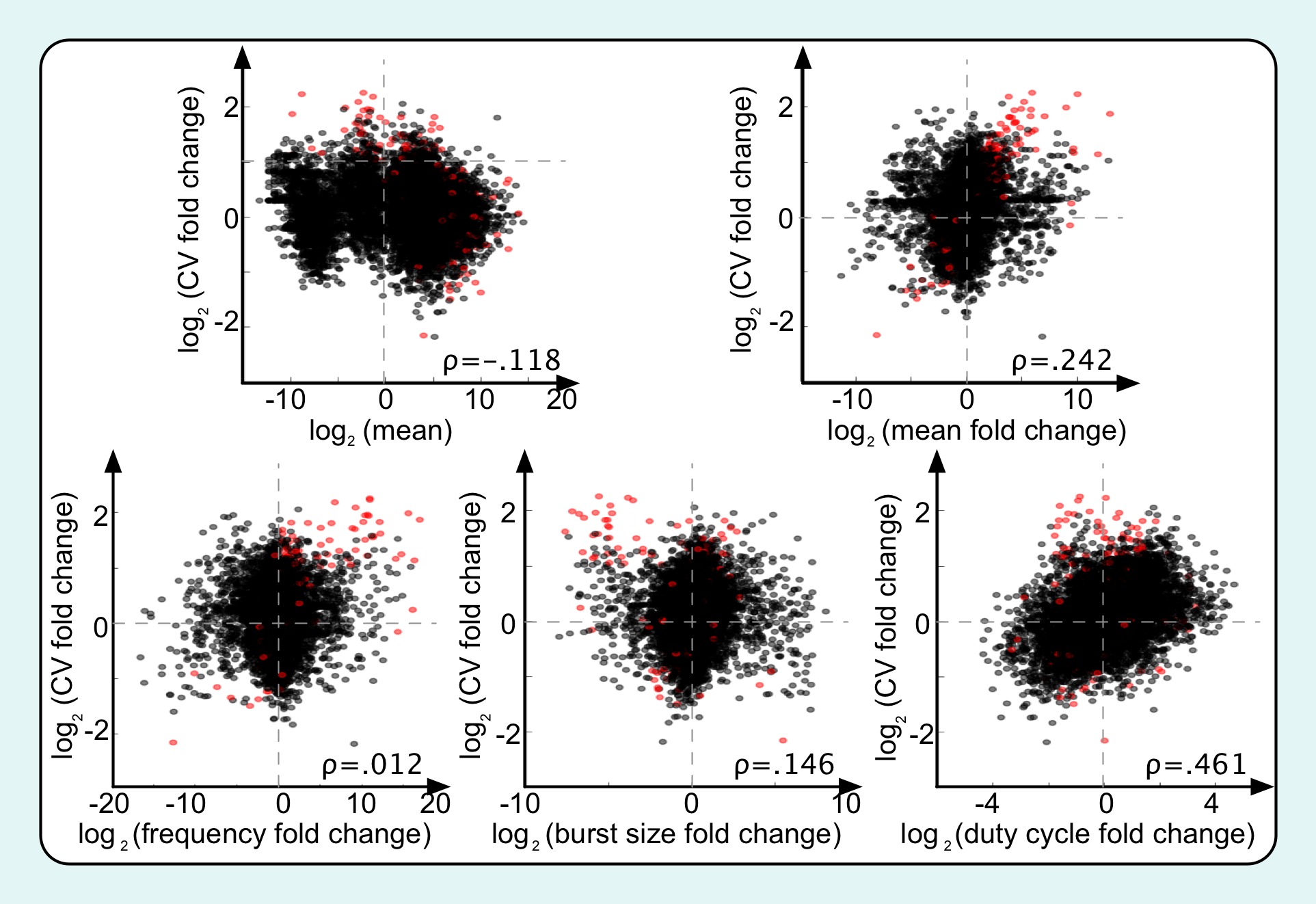

Supplement: Additional file 9 — Figure S6. Scatterplots showing the mean fold-change, as well as the fold-change of the CV compared to the change in burst frequency, duty cycle, burst size for early vs late blastocysts from Deng et al. [10]. In all panels, black dots represent genes which did not change, red dots represent genes which were deemed significant by D3E. (JPEG 345 kb) [file 12859_2016_944_MOESM9_ESM.jpeg]
